# An overview of animal prion diseases

**DOI:** 10.1186/1743-422X-8-493

**Published:** 2011-11-01

**Authors:** Muhammad Imran, Saqib Mahmood

**Affiliations:** 1 Centre for Research in Endocrinology and Reproductive Sciences (CRERS), Department of Physiology and Cell Biology, University of Health Sciences (UHS), Khayaban-e-Jamia Punjab, Lahore 54600, Pakistan; 2 Department of Human Genetics and Molecular Biology, University of Health Sciences (UHS), Khayaban-e-Jamia Punjab, Lahore 54600, Pakistan

**Keywords:** Prion diseases, scrapie, BSE, chronic wasting disease, *PRNP*

## Abstract

Prion diseases are transmissible neurodegenerative conditions affecting human and a wide range of animal species. The pathogenesis of prion diseases is associated with the accumulation of aggregates of misfolded conformers of host-encoded cellular prion protein (PrP^C^). Animal prion diseases include scrapie of sheep and goats, bovine spongiform encephalopathy (BSE) or mad cow disease, transmissible mink encephalopathy, feline spongiform encephalopathy, exotic ungulate spongiform encephalopathy, chronic wasting disease of cervids and spongiform encephalopathy of primates. Although some cases of sporadic atypical scrapie and BSE have also been reported, animal prion diseases have basically occurred via the acquisition of infection from contaminated feed or via the exposure to contaminated environment. Scrapie and chronic wasting disease are naturally sustaining epidemics. The transmission of BSE to human has caused more than 200 cases of variant Cruetzfeldt-Jacob disease and has raised serious public health concerns. The present review discusses the epidemiology, clinical neuropathology, transmissibility and genetics of animal prion diseases.

## Background

Prion diseases are transmissible protein misfolding disorders in which misfolding of a host-encoded prion protein (PrP) occurs. PrP may exist in two forms: a normal cellular prion protein designated as PrP^C ^and a pathogenic misfolded conformer designated as PrP^Sc^. The superscript (Sc) has been used to refer to scrapie, the first and the most ancient animal transmissible spongiform encephalopathy (TSE). Many authors also use superscripts other than (Sc) to distinguish normal and pathogenic (disease-causing) isoforms. These include (res) for resistant and (Dis) for disease. An abbreviated name of a prion disease can also be used as superscript to point out the origin of the pathogenic isoform i.e. PrP^Sc ^or PrP^CJD^. The pathogenic conformers are simply called prions (the infectious protein particles) [1].

According to seeding-nucleation model, the preexisting or acquired PrP^Sc ^oligomers catalyze the conversion of PrP^C ^molecules into PrP^Sc ^fibrils the breakage of which provides more PrP^Sc ^templates for the conversion process. The process of prions' propagation in the brain results in the pathogenesis of prion diseases [2]. Sixteen different variants of prion disease have been reported so far: 9 in humans and 7 in animals. The etiology, host range and year of description for these disease variants are given in Table 1. In the present review, a brief description of animal prion diseases is provided.

**Table 1:** Etiology of prion diseases

Animal prion diseases			
**Disease**	**Host**	**Etiology**	**Year of Description**

Scrapie	Sheep, Goats	Infection with Prions of unknown origin	1732
TME	Mink	Infection with Prions of either sheep or cattle origin	1947
CWD	Cervids	Infection with Prions of unknown origin	1967
BSE	Cattle	Infection with Prions of unknown origin	1986
EUE	Nyala, Kudu	Infection with Prions of BSE origin	1986
FSE	Cats	Infection with prions of BSE origin	1990
NHP	Lemurs	Infection with Prions of BSE origin	1996

**Human prion diseases**			

**Disease**	**Host**	**Etiology**	**Year of Description**

Kuru	Human	Ritualistic Cannibalism or "Transumption"	1900s
sCJD	Human	Spontaneous PrP^C ^→PrP^Sc ^conversion or somatic mutation	1920
fCJD	Human	Mutations in *PRNP*	1924
GSS	Human	Mutations in *PRNP*	1936
iCJD	Human	Infection with Prions of human origin by cadaveric corneal grafts, hGH or dura mater	1974
FFI	Human	*PRNP *haplotype 178N-129M	1986
vCJD	Human	Infection with Prions of BSE origin	1996
sFI	Human	Spontaneous PrP^C ^→PrP^Sc ^conversion or somatic mutation	1999
VPSPr	Human	Spontaneous PrP^C ^→PrP^Sc ^conversion or somatic mutation	2008

TME (transmissible mink encephalopathy), CWD (chronic wasting disease), BSE (bovine spongiform encephalopathy), EUE (exotic ungulate spongiform encephalopathy), FSE (feline spongiform encephalopathy), NHP (TSE in non-human primates), sCJD (sporadic Cruetzfeldt-Jacob disease), fCJD (familial CJD), GSS (Gerstmann-Sträussler-Scheinker syndrome), iCJD (iatrogenic CJD), FFI (fatal familial insomnia), vCJD (variant CJD), sFI (sporadic fatal insomnia), VPSPr (variably protease-sensitive prionopathy)

### Scrapie

Scrapie is the ancient form of TSEs. It is known since 1732 and has occurred in sheep, goats and moufflons [3]. As is the case with other prion diseases, clinicopathological phenotypes of scrapie vary according to the prion strain and animals' genetic background. Multiple prion strains may exist in a single scrapie isolate and a PrP^Sc ^conformer underrepresented in one breed may be selected as dominantly propagating strain in another breed [4,5]. Clinical symptoms may include behavioral changes, blindness, ataxia, incoordination, hyperexitability and tremors. Intense pruritus is the most common symptom which usually leads to wool loss by rubbing and scraping, and results in a characteristic nibbling response from animal when the dorsum is scratched or pressure over the base of tail is applied. The incubation period of scrapie is 2-5 years and death occurs within 2 weeks to 6 months after clinical onset. Neuropathological signs are spongiform vacuolation, astrogliosis and the deposition of PrP^Sc ^amyloid plaques in the central nervous system (CNS). PrP^Sc ^has been detected in the nervous system, tonsils, spleen, lymph nodes, nicitating membrane, muscles, placentas, distal ileum and proximal colon. Detection of PrP^Sc ^in the third eye lid is used as rapid diagnostic test in live animals. Excretions and secretions have also been found to contain PrP^Sc ^infectivity which may transmit between animals horizontally. About 3-5% animals per affected flock may die annually and an increase up to 20% in annual mortality rates has also been noted in some flocks [3,6-9].

The abovementioned facts are for classical or typical sheep scrapie. Atypical cases of both sheep and goat scrapie have also been described. In atypical scrapie, the major clinical symptoms are ataxia and incoordination. Pruritus is uncommon. Clinical signs of goat scrapie overlap with those of sheep scrapie and may include irritability, loss of inquisitiveness, unusual alertness, restlessness, impaired vision, hyperesthesia, incoordination, posture abnormalities, tremors, and teeth grinding, salivation, or regurgitation of rumen contents [10-12].

Several amino acid polymorphisms in both the ovine and caprine PrP encoding genes (*PRNP*) have been reported to be associated with scrapie susceptibility. Sheep exposed to natural or experimental PrP^Sc ^infection have been shown to gain maximum scrapie resistance in the presence of Q171R polymorphism and maximum scrapie susceptibility in the presence of A136V polymorphism. A three codon system based on A136V, R154H and Q171R/H polymorphisms has been developed by using five alleles (ARQ, VRQ, AHQ, ARR and ARH). These five alleles can be combined into 15 genotypes i.e. ARR/ARR or VRQ/ARQ which, in turn, can be classified into five groups (R1-R5) according to the susceptibility they confer to the disease. The most resistant R1 genotype is ARR/ARR and the most susceptible R5 genotypes are VRQ/VRQ, VRQ/ARQ, VRQ/ARH and VRQ/AHQ. The remaining genotypes are of intermediary susceptibility. This risk classification system has helped many European states in selective breeding of sheep against scrapie. The selective breeding of sheep against scrapie caused no considerable negative impacts on production, reproduction and health of animals [13,14]. The presence of ARR/ARR in either ewe or her fetus would resist the PrP^Sc ^accumulation in placenta [7,11]. Though ovine *PRNP *polymorphism has been abundantly linked to the scrapie risk, a few studies have been carried out for the analysis of caprine *PRNP *gene in this regard. This is due to the fact that the prevalence of typical scrapie outbreaks in goat is comparatively much lower than in sheep. However, caprine PrP polymorphisms I142M, H143R, N146S/D, R154H, R211Q and Q222K have been shown to be associated with low scrapie risk. Atypical scrapie is also influenced by variations in *PRNP *and has been reported to occur in animals carrying genotypes conferring resistance to typical scrapie [10-12].

### Transmissible Mink Encephalopathy (TME)

TME is a rare TSE of farmed mink. Mink is a small semi-aquatic wild mammal that is raised in several countries for the production of fur. TME was first recognized in Wisconsin and Minnesota in 1947. Subsequently, TME outbreaks in US have occurred in 60s and 70s with the most recent outbreak in 1985. TME has also been seen in farmed mink in Canada, Finland, East Germany and the former USSR [15]. Although the origin of TME is still unknown, contaminated feed, mainly with the scrapie agent, was presumed to be the main source of infection. Mink inoculated with various strains of the scrapie agent revealed the development of TME. Oral infection of minks with the classical BSE agent also caused TME, but animals exhibited docile rather than aggressive behavior. More recently, the L-type BSE agent has been described as the most likely candidate for being causative of TME [16,17]. TME is readily transmissible to raccoons by parenteral, intracerebral and oral routes, and can intracerebrally be transmitted to striped skunks, ferrets, American sable (pine martens), beech martens, cattle, sheep, goats, hamsters and non-human primates such as rhesus macaque, stump-tailed macaque and squirrel monkey. Non-transgenic mice are not susceptible to TME [17]. TME passaged in cattle has also been transmitted to mink both intracerebrally and orally with incubation periods of only 4-7 months [18]. A bifurcation in clinical phenotypes with distinct incubation periods, neuropathological lesions and biochemical profiles was produced in hamsters on inoculation with TME. Depending on clinical symptoms of the disease, one strain was named as "hyper (HY)" while the other as "drowsy (DY)" [19]. On coinfection of these strains, DY shows a dominant competition for the recruitment of cellular PrP^C ^into oligomers, and may reduce the incubation time or even block the ability of HY to cause the disease [20].

When housed in the same cage, minks may acquire the infection through cannibalism or biting of each other. However, at least in one outbreak, kits housed with their dams did not develop the disease. Environmental exposure and vertical transmission have not been found to cause the spread of the infection. TME has been detected only in adult mink, and may cause 60-90% or even 100% mortality during an outbreak [http://www.cfsph.iastate.edu].

The clinical manifestation of the disease includes behavioral changes such as increased aggressiveness and hyperesthesia, depression, restlessness, and neglect in parental care and coat grooming. The affected minks often soil the nest or scatter feces in the cage. At the earlier disease stages, they may also exhibit difficulty eating and swallowing. Later, symptoms such as abnormal gait, ataxia, incoordination, occasional tremors, clenching of the jaw, curved tail like those of squirrels, and compulsive biting or mutilation of objects or of the self, particularly of the tail, may be noted. Near the end of the disease course, convulsions may occur, and minks become somnolent and unresponsive, and can be seen to press their heads against the cage for hours. Incubation time in naturally occurring TME may range from 6 to 12 months, and death usually occurs within 2 to 8 weeks [17, http://www.cfsph.iastate.edu].

Neuropathological features of TME include extensive spongiform degeneration in the neuropil of the brain. Astrocytosis also occurs. Spongiform changes are intense in the cerebral cortex, particularly in the frontal cortex, as well as the corpus striatum, thalamus and hypothalamus, but are less severe in the midbrain, pons and medulla, and usually, are not evident in the cerebellum and spinal cord. In addition to CNS, PrP deposits of the TME agent, but not amyloid plaques, have been detected in spleen, intestine, the mesenteric lymph node, thymus, kidney, liver and salivary glands of experimentally infected mink [17, http://www.cfsph.iastate.edu].

### Chronic Wasting Disease (CWD)

CWD is a TSE of captive as well as free-ranging members of the family Cervidae. From 1967 to now, CWD has been seen in 14 USA states, 2 Canadian provinces and in imported animals in South Korea. However, surveillance for CWD has been at minimum around the globe. The affected species include mule deer, white-tailed deer, black-tailed deer, Rocky Mountain elk, and Shira's moose. The origin of CWD is still unknown, although intracerebral transmission of the scrapie agent has been shown to induce the disease in elk. More than 1 PrP^CWD ^strains may exist among the affected populations [17,21-23]. Epidemiological and experimental data provide evidence that horizontal transmission of CWD can efficiently occur by contact with affected animals or through environmental exposure [17,22,24-26]. Until now, the natural transmission of CWD has not been evident in humans who have been exposed for long to the affected area and consumed venison, and also not in domestic bovids such as sheep and cattle cosharing the habitat with the affected cervids. Moreover, transgenic mice expressing either the human, ovine or bovine PrP^C ^coding frames did not develop the disease when inoculated with the CWD agent [17,22,26]. However, other cervids such as red deer and reindeer/caribou are also susceptible to CWD through intracerebral and oral routes and may be contributive to sustaining the ongoing CWD epidemic in North America [27,28]. CWD is also intracerebrally transmissible to cattle, sheep, goats, ferrets, hamsters, bank voles, mink, raccoons and squirrel monkeys [22].

The PrP^CWD ^infectivity can be detected in the nervous system, the lymphoreticular system, the hematopoietic system, skeletal and cardiac muscles, pancreas, fat, retina, and the adrenal and salivary glands of naturally and/or experimentally infected animals [17,22,25,29,30]. Virtually, no tissue in infected cervids should be considered free of the CWD agent. Susceptible animals may acquire the infectivity from their habitats during feeding on grasses or by drinking water contaminated with PrP^CWD ^which affected cervids excrete or secrete or deposit into the environment, even in the asymptomatic carrier state, in the form of feces, urine, saliva, blood, placenta and carcasses [17,22,31-33]. Acquisition of the infectivity may be enhanced further by oral abrasions and nasal exposure to PrP^CWD^-containing droplets and aerosols [34,35]. An important point is that the TSE agents can bind to soil particles, persist there for years still retaining the infectivity and transmit the disease via oral route with even more efficiency [36-38]. These data provide a plausible explanation for the high incidence of CWD and efficient transmission of the infectivity among cervids.

Prevalence of the disease in affected herds may range from 0.1 to 50% or even 100%, sometimes. CWD has also been detected in USA in areas far from the original endemic area and raised several questions: Whether the infectivity has been transported to these areas illegally in the form of tainted materials or infected animals or by some another way, whether the scrapie agent has adapted to cause CWD by repeated natural passages into the deer, or if the PrP^C ^conversion in cervids is proficient enough to result in the sporadic emergence of the disease, which may in turn establish the epidemic by horizontal transmission. Given that the routes of PrP^CWD ^transmission are still uncertain, managerial decisions for the disease eradication based on the supervision of animal trade, and quarantine or mandated destruction of effected herds or flocks may appear less promising. Therefore, therapeutic intervention targeting molecular mechanisms involved in the pathogenesis of the disease may be a better substitute for the control of epidemics of CWD and scrapie [22]. In addition, *PRNP *polymorphisms S96G, M132L and S225F in cervids have been associated with resistance to CWD and may, at least in the case of captive animals, be helpful in the disease management by selective breeding [22,30,39-41].

In free-ranging cervids, the increased prevalence of CWD allied with the lower survival of diseased animals is thought to provide predators like mountain lions an opportunity for selective successful predation. Such a selective predation would lead to local imbalances in ecological dynamics of food webs and nutrients recycling [42,43].

Chronic wasting of carcasses or weight loss, on the basis of which the disease is called "chronic wasting disease", is very common in affected cervids. CWD can also cause these animals to have a rough, dry coat, patchy retention of the winter coat in summer. In subclinical or early clinical CWD, affected cervids particularly elk, may also show some other highly subtle symptoms including lassitude, sudden death in deer after handling, a lowered head and drooping ears and behavioral changes such as having fixed gaze and loss of fear of humans. With the progression of the disease, following more perceptible symptoms may arise: flaccid hypotonic facial muscles, ataxia, head tremors, teeth grinding, repetitive walking close to the boundary of the enclosure, hyperexcitability with handling, excessive salivation due to difficulty swallowing, esophageal dilation or ruminal atony, regurgitation of ruminal fluid, polyuria, polydipsia, syncope, and aspiration pneumonia. Many animals become severely emaciated before they die. Incubation periods in CWD lie within the range of 16 months to 5 years and the disease equally affects both males and females. Death usually occurs within a period of 1 year after the onset of clinical signs [17, http://www.cfsph.iastate.edu].

On histopathological examination, CNS of the affected cervids shows intraneuronal vacuolation, degeneration and loss of neurons, extensive neuropil spongiosis, astrocytic hypertrophy and hyperplasia, and occasional amyloid plaques. Spongiform lesions are mainly observed within the thalamus, hypothalamus, midbrain, pons, medulla oblongata, the olfactory tubercle and cortex. The most consistent histological lesions and PrP^CWD ^immunohistochemical staining are seen within the dorsal motor nucleus of the vagus nerve, which is considered the first site of PrP^CWD ^accumulation. Importantly, the clinical signs of polyuria and polydipsia and the low urine specific gravity in clinically dehydrated animals may be attributed to severe lesions in the supra-optic and paraventricular nuclei, where the production of anti-diuretic hormone occurs [17].

### Bovine Spongiform Encephalopathy (BSE or "Mad Cow" disease)

Bovine Spongiform Encephalopathy (BSE) or "the Mad Cow disease" is a progressive and invariably fatal neurodegeneration in cattle. The clinical signs of BSE may include tremors, gait abnormalities particularly of hindlimb (ataxia), aggressive behavior, apprehension, and hyperreactivity to stimuli. PrP^Sc ^accumulation and spongiform vacuolation are usually found in the brain [7]. At the terminal stages of the disease, BSE prions may also be detected in spinal cord, retina, ileum, adrenal glands, tonsils, bone marrow, peripheral nerves, dorsal root ganglia, trigeminal ganglion and thoracic ganglia. The BSE infectivity may be observed in brain tissues as early as 2 years after post inoculation. Epidemiological and transmission studies have found no evidence of BSE prions in milk, semen or embryos and there is little or no evidence of its horizontal transmission. However, the offspring of infected animals have shown an increased risk for disease development. The incubation period for BSE is 2 to 8 years and most of BSE cases have been found in 4 to 5 years old dairy cattle [[7], http://www.cfsph.iastate.edu]. Apart from BSE strain causing classical BSE, two other strains (H-type and L-type) causing atypical BSE have been described. Most of atypical BSE cases have been detected during active surveillance targeting fallen stocks and slaughtered animals [44,45]. Amyloid plaques can be detected in animals with atypical L-form BSE, although they are not typical of classical BSE [http://www.cfsph.iastate.edu].

BSE cases have occurred worldwide in nearly 0.2 million Holstein-Freisian *Bos taurus *cattle, 1 *Bos indicus *animal, and 1 *Bos taurus *× *Bos indicus *cow [7,44,46-48]. BSE first appeared in mid 80s in UK, soon evolved to epidemic proportions with 1000 cases occurring per week in 1992, and has been shown to be naturally transmissible to a number of zoo species [17]. BSE has also transmitted to humans in the form of a variant of Cruetzfeldt-Jacob disease (vCJD) [49]. The practice of using meat and bone meal (MBM) possibly contaminated with infectious mammalian pathogenic prions in cattle feed was considered the likely cause of the BSE epidemic. A ban on the use of MBM in the ruminants feed ultimately resulted in a progressive decline of the epidemic [44]. Natural cases of transmissions of BSE have been described in sheep and goats. Such bovid animals may be an additional source of BSE transmission to humans [50,51]. Indeed, transgenic mice expressing human PrP^C ^are more susceptible to sheep-passaged BSE than classical BSE [52]. Origins of BSE are unknown. The accidental inclusion of tissues in MBM from familial or sporadic BSE cases, sheep with scrapie or CJD patients may be responsible [46,47,53-56].

Although several *PRNP *variants have been described for cattle [57,58], only three amongst these variants, a 23 bp indel in the *PRNP *promoter, a 12 bp indel in the first intron and an E211K polymorphism, are known to confer susceptibility to BSE [46,47,58-60].

### Exotic Ungulate Spongiform Encephalopathy (EUE)

EUE is a TSE of exotic zoo ruminants of the family Bovidae. During a period overlapping with BSE epidemic, 6 greater kudu, 6 elands, 2 each of Arabian oryx and ankole cattle, and 1 each of gemsbok, nyala, scimitar-horned oryx and bison were diagnosed with EUE from UK. The affected animals had been fed meat and bone meal (MBM) derived from ruminants. Indeed, mice inoculated with brain homogenates from greater kudu and nyala with EUE and from cattle with BSE developed a TSE with similar profiles of neuropathological lesions and incubation periods. Strain typing studies in these mice also revealed a similarity between the EUE and BSE strains, supporting the hypothesis that EUE is caused by an infection with PrP^BSE^. The course of EUE and clinical symptoms varies, according to the species, and are distinct from those of BSE and scrapie. All EUE cases died of this disease [17,61,62].

### Feline Spongiform Encephalopathy (FSE)

FSE is a TSE of domestic cats and captive wild members of the family Felidae. Since 1990 to present, nearly 100 domestic cats usually from UK including 1 case each from Northern Ireland, Norway, Liechtenstein and Switzerland, and 29 captive wild cats including 15 cheetahs, 4 lions, 3 each of ocelots, pumas, tigers and an Asian golden cat have been diagnosed with FSE. FSE cases in wild cats have been seen in zoos of UK, France, Australia, Ireland, and Germany. All large zoo cats diagnosed with FSE outside UK originated from UK except a female cheetah and her cub born in France and another thought to have acquired the infectivity in Netherland where she was born. As most of the FSE cases occurred in parallel to the BSE epidemic, exposure of affected cats to feed contaminated with PrP^BSE ^was taken as causative of the disease. Mice inoculated with brain homogenates from cats with FSE and cattle with BSE developed a TSE with similar profiles of neuropathological lesions and incubation periods. Strain typing studies in these mice also revealed a similarity between the FSE and BSE strains, supporting the hypothesis that FSE is caused by an infection with BSE prions [17,63,64]. However, in 1998, a domestic cat and his owner have been shown to be affected with a similar strain distinct from PrP^BSE^. A bifurcation in phenotypes was noted: the man revealed a phenotype reminiscent of sCJD rather than of vCJD and the cat showed a clinical phenotype distinct from FSE. It remains unknown whether this incidence was due to a chance, whether a horizontal transmission occurred between the man and the cat, or if both contracted the disease from the same unknown source [65]. Another cat infected with such strain and the acquisition of FSE from the environment has not been reported yet. With a ban on the use as pet feed of bovine spleen and CNS tissue, the FSE epidemic declined rapidly. All FSE cases have been seen in cats aged more than 2 years [17].

The clinical manifestation of the disease includes severe behavioral changes, depression, restlessness and neglect in coat grooming. The behavioral changes include fear, uncharacteristic aggressiveness or unusual timidity and hiding. Abnormal or hypermetric gait and ataxia, mainly of the hind limbs, are also characteristics of FSE. Affected cats often show poor judgment of distance and hyperesthesia to touch or noise, and may also develop tremors, stare vacantly or circle. In addition, excessive salivation, polyphagia, polydipsia and dilated pupils have been reported as symptoms of FSE. At the end of the disease course, convulsions may occur and somnolence is common. Death occurs after 3-8 and 8-10 weeks of clinical onset of the disease in domestic cats and cheetahs, respectively [[17], http://www.cfsph.iastate.edu].

Neuropathological profiles of FSE include spongiform degeneration in the neuropil of the brain and spinal cord, with a predominance of the most severe lesions in the medial geniculate nucleus of the thalamus and the basal nuclei. PrP deposits of the FSE agent, with characteristic florid plaques, have been detected by immunohistochemistry in the central and peripheral nervous system, retina, lymphoreticular system, kidney and adrenal glands [[17,63,64], http://www.cfsph.iastate.edu).

### TSE in non-human primates (NHP)

Two Mayotte brown lemurs and 1 each of white fronted brown lemur, mongoose lemur and Rhesus macaque from a zoo and three primate facilities in France were diagnosed with a TSE, from 1996 to 1999. Although a large number of non-human primates are susceptible to experimental exposure to various TSE strains, they have never been reported before this to contract a TSE naturally. The affected animals had been fed primate diets likely contaminated with meat from UK. Indeed, lemurs experimentally inoculated with brain homogenates from cattle with BSE developed a TSE with profiles of neuropathological lesions similar to those seen in naturally infected lemurs. Strain typing studies also revealed a similarity between the two strains. Further, in these naturally and experimentally infected lemurs, immunohistochemical examination showed similar staining patterns and the distribution of PrP^res ^in the brain, spinal cord, tonsils, spleen and various sections of the gut and gut-associated lymphatic tissues [17,66].

## Conclusion

Animal prion diseases other than scrapie and CWD have been controlled by the withdrawal of source of contamination from animals' diet. However, scrapie and CWD are self-sustaining epidemics and their control necessitates the development of therapeutics that can block the cellular spread of infectivity or the propagation of prions [22]. No case of prion disease has been observed from Pakistan up till now. We have recently performed the analysis of the bovine *PRNP *in 236 Pakistani cattle representing 7 different breeds. The results suggest that Pakistani cattle are genetically more resistant to BSE as compared to European cattle (unpublished data).

## Competing interests

The authors declare that they have no competing interests.

## Authors' contributions

MI^1 ^surveyed literature and wrote the article. SM^2 ^organized the contents and submitted the article. All authors read and approved the final manuscript.

## References

[B1] Prusiner SB Novel proteinaceous infectious particles cause scrapie Science 1982 216 136 144 10.1126/science.6801762 6801762

[B2] Collinge J Clarke AR A general model of prion strains and their pathogenicity Science 2007 318 930 936 10.1126/science.1138718 17991853

[B3] Jeffrey M González L Classical sheep transmissible spongiform encephalopathies: pathogenesis, pathological phenotypes and clinical disease Neuropathol Appl Neurobiol 2007 33 373 394 10.1111/j.1365-2990.2007.00868.x 17617870

[B4] Thackray AM Hopkins L Lockey R Spiropoulos J Bujdoso R Emergence of multiple prion strains from single isolates of ovine scrapie J Gen Virol 2011 92 1482 1491 10.1099/vir.0.028886-0 21270287

[B5] Yokoyama T Masujin K Schmerr MJ Shu Y Okada H Iwamaru Y Imamura M Matsuura Y Murayama Y Mohri S Intraspecies prion transmission results in selection of sheep scrapie strains PLoS One 2010 5 e15450 10.1371/journal.pone.0015450 21103326 PMC2982847

[B6] Maddison BC Rees HC Baker CA Taema M Bellworthy SJ Thorne L Terry LA Gough KC Prions are secreted into the oral cavity in sheep with preclinical scrapie J Infect Dis 2010 201 1672 1676 10.1086/652457 20402590

[B7] Novakofski J Brewer MS Mateus-Pinilla N Killefer J McCusker RH Prion biology relevant to bovine spongiform encephalopathy J Anim Sci 2005 83 1455 1476 15890824 10.2527/2005.8361455x

[B8] O'Rourke KI Zhuang D Truscott TC Yan H Schneider DA Sparse PrP-Sc accumulation in the placentas of goats with naturally acquired scrapie BMC Vet Res 2011 7 7 10.1186/1746-6148-7-7 21284878 PMC3041672

[B9] van Keulen LJM Bossers A van Zijderveld F TSE pathogenesis in cattle and sheep Vet Res 2008 39 24 10.1051/vetres:2007061 18258167

[B10] Benestad SL Arsac JN Goldmann W Nöremark M Atypical/Nor98 scrapie: properties of the agent, genetics, and epidemiology Vet Res 2008 39 19 10.1051/vetres:2007056 18187032

[B11] Goldmann W PrP genetics in ruminant transmissible spongiform encephalopathies Vet Res 2008 39 30 10.1051/vetres:2008010 18284908

[B12] Vaccari G Panagiotidis CH Acin C Peletto S Barillet F Acutis P Bossers A Langeveld J van Keulen L Sklaviadis T Badiola JJ Oletti OA Groschup MH Agrimi U Foster J Goldmann W State-of-the-art review of goat TSE in the European Union, with special emphasis on PRNP genetics and epidemiology Vet Res 2009 40 48 10.1051/vetres/2009031 19505422 PMC2704333

[B13] Dawson M Moore RC Bishop SC Progress and limits of PrP gene selection policy Vet Res 2008 39 25 10.1051/vetres:2007064 18258168

[B14] Sweeney T Hanrahan JP The evidence of associations between prion protein genotype and production, reproduction, and health traits in sheep Vet Res 2008 39 28 10.1051/vetres:2008004 18284907

[B15] Marsh RF Hadlow WJ Transmissible mink encephalopathy Rev Sci Tech 1992 11 539 550 1535524 10.20506/rst.11.2.606

[B16] Baron T Bencsik A Biacabe AG Morignat E Bessen RA Phenotypic similarity of transmissible mink encephalopathy in cattle and L-type bovine spongiform encephalopathy in a mouse model Emerg Infect Dis 2007 13 1887 1894 18258040 10.3201/eid13112.070635 PMC2876762

[B17] Sigurdson CJ Miller MW Other animal prion diseases Brit Med Bull 2003 66 199 212 10.1093/bmb/66.1.199 14522860

[B18] Marsh RF Bessen RA Lehmann S Hartsough GR Epidemiological and experimental studies on a new incident of transmissible mink encephalopathy J Gen Virol 1991 72 589 594 10.1099/0022-1317-72-3-589 1826023

[B19] Bessen RA Marsh RF Identification of two biologically distinct strains of transmissible mink encephalopathy in hamsters J Gen Virol 1992 73 329 234 10.1099/0022-1317-73-2-329 1531675

[B20] Shikiya RA Ayers JI Schutt CR Kincaid AE Bartz JC Coinfecting prion strains compete for a limiting cellular resource J Virol 2010 84 5706 5714 10.1128/JVI.00243-10 20237082 PMC2876617

[B21] Angers RC Kang HE Napier D Browning S Seward T Mathiason C Balachandran A McKenzie D Castilla J Soto C Jewell J Graham C Hoover EA Telling GC Prion strain mutation determined by prion protein conformational compatibility and primary structure Science 2010 328 1154 1158 10.1126/science.1187107 20466881 PMC4097672

[B22] Sigurdson CJ A prion disease of cervids: Chronic wasting disease Vet Res 2008 39 41 10.1051/vetres:2008018 18381058

[B23] Tamgüney G Miller MW Giles K Lemus A Glidden DV DeArmond SJ Prusiner SB Transmission of scrapie and sheep-passaged bovine spongiform encephalopathy prions to transgenic mice expressing elk prion protein J Gen Virol 2009 90 1035 1047 10.1099/vir.0.007500-0 19264659 PMC2804060

[B24] Mathiason CK Hays SA Powers J Hayes-Klug J Langenberg J Dahmes SJ Osborn DA Miller KV Warren RJ Mason GL Hoover EA Infectious prions in pre-clinical deer and transmission of chronic wasting disease solely by environmental exposure PLoS One 2009 4 e5916 10.1371/journal.pone.0005916 19529769 PMC2691594

[B25] Seelig DM Mason GL Telling GC Hoover EA Pathogenesis of chronic wasting disease in cervidized transgenic mice Am J Pathol 2010 176 2785 2797 10.2353/ajpath.2010.090710 20395435 PMC2877840

[B26] Tamgüney G Giles K Bouzamondo-Bernstein E Bosque PJ Miller MW Safar J DeArmond SJ Prusiner SB Transmission of elk and deer prions to transgenic mice J Virol 2006 80 9104 9114 10.1128/JVI.00098-06 16940522 PMC1563923

[B27] Balachandran A Harrington NP Algire J Soutyrine A Spraker TR Jeffrey M González L O'Rourke KI Experimental oral transmission of chronic wasting disease to red deer (Cervus elaphus elaphus): early detection and late stage distribution of protease-resistant prion protein Can Vet J 2010 51 169 178 20436863 PMC2808282

[B28] Martin S Jeffrey M González L Sisó S Reid HW Steele P Dagleish MP Stack MJ Chaplin MJ Balachandran A Immunohistochemical and biochemical characteristics of BSE and CWD in experimentally infected European red deer (Cervus elaphus elaphus) BMC Vet Res 2009 5 26 10.1186/1746-6148-5-26 19635142 PMC2724422

[B29] Race B Meade-White K Race R Chesebro B Prion infectivity in fat of deer with chronic wasting disease J Virol 2009 83 9608 9610 10.1128/JVI.01127-09 19570855 PMC2738259

[B30] Spraker TR O'Rourke KI Gidlewski T Powers JG Greenlee JJ Wild MA Detection of the abnormal isoform of the prion protein associated with chronic wasting disease in the optic pathways of the brain and retina of Rocky Mountain elk (Cervus elaphus nelsoni) Vet Pathol 2010 47 536 546 10.1177/0300985810363702 20382822

[B31] Nichols TA Pulford B Wyckoff AC Meyerett C Michel B Gertig K Hoover EA Jewell JE Telling GC Zabel MD Detection of protease-resistant cervid prion protein in water from a CWD-endemic area Prion 2009 3 171 183 10.4161/pri.3.3.9819 19823039 PMC2802782

[B32] Safar JG Lessard P Tamgüney G Freyman Y Deering C Letessier F Dearmond SJ Prusiner SB Transmission and detection of prions in feces J Infect Dis 2008 198 81 89 10.1086/588193 18505383 PMC2803675

[B33] Tamgüney G Miller MW Wolfe LL Sirochman TM Glidden DV Palmer C Lemus A DeArmond SJ Prusiner SB Asymptomatic deer excrete infectious prions in faeces Nature 2009 461 529 532 10.1038/nature08289 19741608 PMC3186440

[B34] Denkers ND Seelig DM Telling GC Hoover EA Aerosol and nasal transmission of chronic wasting disease in cervidized mice J Gen Virol 2010 91 1651 1658 10.1099/vir.0.017335-0 20164261 PMC2888164

[B35] Denkers ND Telling GC Hoover EA Minor oral lesions facilitate transmission of chronic wasting disease J Virol 2011 85 1396 1399 10.1128/JVI.01655-10 21084472 PMC3020502

[B36] Johnson CJ Phillips KE Schramm PT McKenzie D Aiken JM Pedersen JA Prions adhere to soil minerals and remain infectious PLoS Pathog 2006 2 e32 10.1371/journal.ppat.0020032 16617377 PMC1435987

[B37] Johnson CJ Pedersen JA Chappell RJ McKenzie D Aiken JM Oral transmissibility of prion disease is enhanced by binding to soil particles PLoS Pathog 2007 3 e93 10.1371/journal.ppat.0030093 17616973 PMC1904474

[B38] Seidel B Thomzig A Buschmann A Groschup MH Peters R Beekes M Terytze K Scrapie agent (Strain 263K) can transmit disease via the oral route after persistence in soil over years PLoS ONE 2007 2 e435 10.1371/journal.pone.0000435 17502917 PMC1855989

[B39] Green KM Castilla J Seward TS Napier DL Jewell JE Soto C Telling GC Accelerated high fidelity prion amplification within and across prion species barriers PLoS Pathog 2008 4 e1000139 10.1371/journal.ppat.1000139 18769716 PMC2516356

[B40] O'Rourke KI Spraker TR Zhuang D Greenlee JJ Gidlewski TE Hamir AN Elk with a long incubation prion disease phenotype have a unique PrPd profile Neuroreport 2007 18 1935 1938 10.1097/WNR.0b013e3282f1ca2f 18007190

[B41] White SN O'Rourke KI Gidlewski T VerCauteren KC Mousel MR Phillips GE Spraker TR Increased risk of chronic wasting disease in Rocky Mountain elk associated with decreased magnesium and increased manganese in brain tissue Can J Vet Res 2010 74 50 53 20357959 PMC2801312

[B42] Krumm CE Conner MM Hobbs NT Hunter DO Miller MW Mountain lions prey selectively on prion-infected mule deer Biol Lett 2010 6 209 211 10.1098/rsbl.2009.0742 19864271 PMC2865069

[B43] Miller MW Swanson HM Wolfe LL Quartarone FG Huwer SL Southwick CH Lukacs PM Lions and prions and deer demise PLoS One 2008 3 e4019 10.1371/journal.pone.0004019 19107193 PMC2602978

[B44] Ducrot C Arnold M de Koeijer A Heim D Calavas D Review on the epidemiology and dynamics of BSE epidemics Vet Res 2008 39 15 10.1051/vetres:2007053 18187031

[B45] Thiry E Saegerman C Xambeu L Penders J Current status of transmissible spongiform encephalopathies in ruminants Biotechnol Agron Soc Environ 2004 8 221 228

[B46] Nicholson EM Brunelle BW Richt JA Kehrli ME Jr Greenlee JJ Identification of a heritable polymorphism in bovine PRNP associated with genetic transmissible spongiform encephalopathy: evidence of heritable BSE PLoS ONE 2008 3 e2912 10.1371/journal.pone.0002912 18698343 PMC2488391

[B47] Richt JA Hall SM BSE case associated with prion protein gene mutation PLoS Pathog 2008 4 e1000156 10.1371/journal.ppat.1000156 18787697 PMC2525843

[B48] Seuberlich T Botteron C Wenker C Café-Marçal V Oevermann A Haase B Leeb T Heim D Zurbriggen A Spongiform encephalopathy in a miniature zebu Emerg Infect Dis 2006 12 1950 1953 10.3201/eid1212.060750 17326950 PMC3291368

[B49] Bruce ME Will RG Ironside JW McConnell I Drummond D Suttie A McCardle L Chree A Hope J Birkett C Cousens S Fraser H Bostock CJ Transmissions to mice indicate that 'new variant' CJD is caused by the BSE agent Nature 1997 389 498 501 10.1038/39057 9333239

[B50] Houston F Foster JD Chong A Hunter N Bostock CJ Transmission of BSE by blood transfusion in sheep Lancet 2000 356 999 1000 10.1016/S0140-6736(00)02719-7 11041403

[B51] Vulin J Biacabe AG Cazeau G Calavas D Baron T Molecular typing of protease-resistant prion protein in transmissible spongiform encephalopathies of small ruminants, France, 2002-2009 Emerg Infect Dis 2011 17 55 63 21192855 10.3201/eid1701.100891 PMC3204636

[B52] Plinston C Hart P Chong A Hunter N Foster J Piccardo P Manson JC Barron RM Increased susceptibility of human-PrP transgenic mice to bovine spongiform encephalopathy following passage in sheep J Virol 2011 85 1174 1181 10.1128/JVI.01578-10 21084466 PMC3020518

[B53] Baron T Vulin J Biacabe AG Lakhdar L Verchere J Torres JM Bencsik A Emergence of classical BSE strain properties during serial passages of H-BSE in wild-type mice PLoS One 2011 6 e15839 10.1371/journal.pone.0015839 21264286 PMC3021503

[B54] Capobianco R Casalone C Suardi S Mangieri M Miccolo C Limido L Catania M Rossi G Fede GD Giaccone G Bruzzone MG Minati L Corona C Acutis P Gelmetti D Lombardi G Groschup MH Buschmann A Zanusso G Monaco S Caramelli M Tagliavini F Conversion of the BASE prion strain into the BSE strain: The origin of BSE? PLoS Pathog 2007 3 e31 10.1371/journal.ppat.0030031 17352534 PMC1817656

[B55] Colchester AC Colchester NT The origin of bovine spongiform encephalopathy: The human prion disease hypothesis Lancet 2005 366 856 861 10.1016/S0140-6736(05)67218-2 16139661

[B56] Hill AF Sidle KC Joiner S Keyes P Martin TC Dawson M Collinge J Molecular screening of sheep for bovine spongiform encephalopathy Neurosci Lett 1998 255 159 162 10.1016/S0304-3940(98)00736-8 9832197

[B57] Seabury CM Honeycutt RL Rooney AP Halbert ND Derr JN Prion protein gene (PRNP) variants and evidence for strong purifying selection in functionally important regions of bovine exon 3 Proc Natl Acad Sci USA 2004 101 15142 15147 10.1073/pnas.0406403101 15477588 PMC524052

[B58] Sander P Hamann H Pfeiffer I Wemheuer W Brenig B Groschup MH Ziegler U Distl O Leeb T Analysis of sequence variability of the bovine prion protein gene (PRNP) in German cattle breeds Neurogenetics 2004 5 19 25 10.1007/s10048-003-0171-y 14727152

[B59] Heaton MP Keele JW Harhay GP Richt JA Koohmaraie M Wheeler TL Shackelford SD Casas E King DA Sonstegard TS Van Tassell CP Neibergs HL Chase CC Jr Kalbfleisch TS Smith TPL Clawson ML Laegreid WW Prevalence of the prion protein gene E211K variant in U.S. cattle BMC Vet Res 2008 4 25 10.1186/1746-6148-4-25 18625065 PMC2478677

[B60] Juling K Schwarzenbacher H Williams JL Fries R A major genetic component of BSE susceptibility BMC Biology 2006 4 33 10.1186/1741-7007-4-33 17014722 PMC1601964

[B61] Kirkwood JK Cunningham AA Epidemiological observations on spongiform encephalopathies in captive wild animals in the British Isles Vet Rec 1994 135 296 303 10.1136/vr.135.13.296 7817514

[B62] Kirkwood JK Cunningham AA Wells GA Wilesmith JW Barnett JE Spongiform encephalopathy in a herd of greater kudu (Tragelaphus strepsiceros): epidemiological observations Vet Rec 1993 133 360 364 10.1136/vr.133.15.360 8256421

[B63] Bencsik A Debeer S Petit T Baron T Possible case of maternal transmission of feline spongiform encephalopathy in a captive cheetah PLoS One 2009 4 e6929 10.1371/journal.pone.0006929 19738899 PMC2732902

[B64] Eiden M Hoffmann C Balkema-Buschmann A Müller M Baumgartner K Groschup MH Biochemical and immunohistochemical characterization of feline spongiform encephalopathy in a German captive cheetah J Gen Virol 2010 91 2874 2883 10.1099/vir.0.022103-0 20660146

[B65] Zanusso G Nardelli E Rosati A Fabrizi G Ferrari S Carteri A De Simone F Rizzuto N Monaco S Simultaneous occurrence of spongiform encephalopathy in a man and his cat in Italy Lancet 1998 352 1116 1117 10.1016/S0140-6736(05)79756-7 9798590

[B66] Bons N Mestre-Frances N Belli P Cathala F Gajdusek DC Brown P Natural and experimental oral infection of nonhuman primates by bovine spongiform encephalopathy agents Proc Natl Acad Sci USA 1999 96 4046 4051 10.1073/pnas.96.7.4046 10097160 PMC22417

